# Human C-peptide Dose Dependently Prevents Early Neuropathy in the BB/Wor-rat

**DOI:** 10.1155/EDR.2001.187

**Published:** 2001

**Authors:** W. Zhang, M. Yorek, C. R. Pierson, Y. Murakawa, A. Breidenbach, A. A. F. Sima

**Affiliations:** 1 Department of Pathology Wayne State University 540 E. Canfield Ave Detroit MI 48201 USA; 2 Department of Neurotogy Wayne State University Detroit MI USA; 3 Morris Hood Jr. Diabetes Center Wayne State University Detroit MI USA; 4 Department of Internal Medicine University of Iowa Iowa IO USA; 5 Schwarz Pharma AG Monheim Germany

## Abstract

In order to explore the neuroprotective and crossspecies activities of.C-peptide on type 1 diabetic neuropathy, spontaneously diabetic BB/W-rats were given increasing doses of human recombinant Cpeptide (hrC-peptide). Diabetic rats received 10, 100, 500, or 1000 μg of hrC-peptide/kg body weight/ day from onset of diabetes. After 2 months of hrC-peptide administration, 100 μg and greater doses completely prevented the nerve conduction defect, which was associated with a significant but incomplete prevention of neural Na^+^/K^+^-ATPase activity in diabetic rats with 500 μg or greater C-peptide replacement. Increasing doses of hrC-peptide showed increasing prevention of early structural abnormalities such as paranodal swelling and axonal degeneration and an increasing frequency of regenerating sural nerve fibers. We conclude that hrC-peptide exerts a dose dependent protection on type 1 diabetic neuropathy in rats and that this effect is probably mediated by the partially conserved sequence of the active C-terminal pentapeptide


*Int. Jnl. Experimental Diab. Res.*, Vol. 2, pp. 187–193
Reprints available directly from the publisher
Photocopying permitted by license only

© 2001 OPA (Overseas Publishers Association) N.V.
Published by license under
the Harwood Academic Publishers imprint,
part of Gordon and Breach Publishing,
a member of the Taylor & Francis Group.
Printed in the U.S.A.

### Human C-peptide Dose Dependently Prevents Early Neuropathy in the BB/Wor-rat

W. ZHANG[1,3], M. YOREK[4], C. R. PIERSON[1,3], Y. MURAKAWA[1,3], A. BREIDENBACH[5], A. A. F. SIMA[1,2,3]

*Departments of [1]Pathology, [2]Neurology and [3]Morris Hood Jr. Diabetes Center, Wayne State University, Detroit, MI, [4]Department of Internal Medicine, University of Iowa, Iowa City, IO and [5]Schwarz Pharma AG, Monheim, Germany*

*(Received 11 April 2001; Revised 31 May 2001; In final form 2 July 2001)*

**In order to explore the neuroprotective and cross-species activities of C-peptide on type 1 diabetic neuropathy, spontaneously diabetic BB/W-rats were given increasing doses of human recombinant C-peptide (hrC-peptide). Diabetic rats received 10, 100, 500, or 1000 μg of hrC-peptide/kg body weight/day from onset of diabetes. After 2 months of hrC-peptide administration, 100 μg and greater doses completely prevented the nerve conduction defect, which was associated with a significant but incomplete prevention of neural $Na^+/K^+$-ATPase activity in diabetic rats with 500 μg or greater C-peptide replacement. Increasing doses of hrC-peptide showed increasing prevention of early structural abnormalities such as paranodal swelling and axonal degeneration and an increasing frequency of regenerating sural nerve fibers. We conclude that hrC-peptide exerts a dose dependent protection on type 1 diabetic neuropathy in rats and that this effect is probably mediated by the partially conserved sequence of the active C-terminal pentapeptide.**

*Keywords*: Human C-peptide, BB/Wor-rat, diabetic neuropathy, prevention.

### INTRODUCTION

There is now mounting evidence demonstrating that C-peptide has preventive and ameliorating effects on the chronic complications of type 1 diabetes in both humans and experimental animal models [1-3].

In a recent study [4], we demonstrated the preventive and therapeutic effects of rat II C-peptide on the acute and chronic metabolic, functional and structural abnormalities of diabetic polyneuropathy (DPN) in type 1 diabetic BB/Wor-rat. Although, it is not clear how C-peptide exerts these beneficial effects, recent studies suggest that C-peptide has insulinomimetic metabolic and mitogenic effects [5-7]. A specific C-peptide receptor has not yet been characterized [8]. The metabolic effects of C-peptide include corrective effects on nerve $Na^+/K^+$-ATPase and endothelial NO [7-10], which has been associated with its beneficial effects on peripheral nerve function in experimental type 1 diabetic rat models and humans [1,4,10]. C-peptide has by itself no effect on hyperglycemia [4,11].

It has been proposed that the mid-portion of human C-peptide mediates all or part of its biological activity [1], since it is highly conserved in mammalian species. On the other hand, the C-terminal pentapeptide, which possesses receptor/ligand type interaction shows the same biological activity as the entire 31 amino acid C-peptide [8,11]. In the present study we examined the dose response relationship between four doses of human recombinant C-peptide (hrC-peptide) given by subcutaneous osmotic pumps on neural

Address correspondence to: Anders A.F. Sima, MD., Ph.D Department of Pathology, Wayne State University, 540 E. Canfield Ave, Detroit, MI 48201 Phone: (313)-577-1103, Fax : (313)-577-0057, e-mail: asima@med.wayne.edu.

$Na^+/K^+$-ATPase, nerve conduction velocity (NCV) and early structural changes in two month diabetic BB/Wor-rats.

### MATERIALS AND METHODS

### Animals

Fifty prediabetic male BB/Wor-rats and ten age- and sex-matched non-diabetes prone BB/Wor-rats were obtained from Biomedical Research Models (Worcester, MA, U.S.A.). All animals were maintained in air-filtered metabolic cages with ad libitum access to rat chow (Wayne lab blox F-6, Wayne Food Division, Chicago, IL, U.S.A.) and water. Body weight and urine glucose were monitored daily to ascertain the onset ($76 \pm 4$ days of age) of diabetes, following which diabetic animals were given daily titrated doses (0.5-3.5 I.U.) of protamine zinc insulin (Blue Ridge Pharmaceuticals, NC, U.S.A.) in order to maintain blood glucose levels between 20-25 mmol/l and to prevent ketoacidosis as previously described [4]. Blood glucose was measured every two weeks and at the end of the study. Immediately after onset of diabetes, rats were randomly assigned to five treatment groups, eight animals per group, diabetic rats were either sham operated or received 10, 100, 500, or 1000 μg hrC-peptide per kg/day (Schwarz Pharma AG, Monheim, Germany). HrC-peptide was dissolved in phosphate-buffered saline and delivered via subcutaneously implanted osmotic pumps (Alza Corporation, Palo Alto, CA, U.S.A.). Non-diabetic control animals ($n = 10$) were sham operated.

### Electrophysiological Studies

Nerve conduction velocity was measured at 1 and 2 weeks and 1 and 2 months of diabetes. It was measured in the left sciatic-tibial nerves under temperature controlled (35°-37°C) conditions [12]. The left sciatic nerve was stimulated supramaximally (8V) with square wave pulses (20Hz) at the sciatic notch and the tibial nerve at the ankle using an electromyography machine (5200 A, Cadwell Laboratory, Kennewick, WA, U.S.A.). The compound evoked motor responses were obtained from the first interosseous space and were measured from stimulus artifact to onset of the M-wave deflection [12]. Each NCV value represented the averaging of 8 or 16 recordings and was calculated by subtracting the distal from the proximal latency divided by the distance between the two stimulating electrodes, giving NCV in $m.sec^{-1}$.

### Tissue Collection

Non-fasted animals were sacrificed with a Na-pentobarbital overdose (100 mg/kg body wt. i.p.) and both sciatic nerves were dissected, weighed and snap-frozen in liquid nitrogen and stored at −70°C for measurement of $Na^+/K^+$-ATPase activity. The right sural nerve was fixed in situ with 2.5% glutaraldehyde in 0.1 M cacodylate buffer (pH 7.40), dissected and immersion fixed in the same fixative overnight at 4°C and post-fixed in cacodylate buffered (0.1 M) 1% osmium tetroxide (pH 7.40) overnight at 4°C. The sural nerve was dehydrated and single myelinated fibers were teased in unpolymerized Epon as previously described [4,12]. Plasma was collected for assessment of hrC-peptide levels, which were determined by HPLC-Electrospray Mass Spectroscopy.

### Biochemical Analysis

For assessment of $Na^+/K^+$-ATPase activity, nerve samples were homogenized in 2 ml of 0.2 M sucrose and 0.02 M Tris-HCl at pH 7.5. Ten to 20 μl of the homogenate was assayed enzymatically for total ATPase activity in 1 ml of 100 mM NaCl, 10 mM KCl, 2.5 mM $MgCl_2$, 1 mM ATP, 1 mM phosphoenolpyruvate, 30 mM imidazole HCl buffer (pH 7.3), 0.15 mM NADH, 50 μg lactate dehydrogenase and 30 μg pyruvate kinase [13]. To measure ouabain-inhibitable ATPase activity, 20 μl of 25 mM ouabain was added. $Na^+/K^+$-ATPase activity was defined as the difference in activity before and after addition of ouabain and was expressed as μmol ADP formed per gram of wet weight per hour. Assays were performed in duplicates.

### Teased Fiber Examination

A mean of 168 ± 4 myelinated fibers were teased from each sural nerve and scored for specific changes, providing a three dimentional assessment of myelinated fiber pathology as previously described in detail [4,12]. Representing the temporal sequence and increasing severity of myelinated fiber pathology, they were classified as follows: normality, paranodal swelling, paranodal demyelination, excessive myelin wrinkling, intercalated internodes, segmental demyelination, Wallerian degeneration, and regeneration. Each fiber was scored as to its most severe change and expressed as a percentage of total fibers.

### Statistical Analysis

The results are presented as mean ± SD and the significance of differences was calculated by analysis of variance (ANOVA) with SPSS version 10.0.7 software. When an overall difference of $p < 0.05$ was obtained, group differences were assessed by post hoc analysis using Scheffe's test. Tissue samples for biochemical and teased fiber analysis were coded in order to mask animal identity.

### RESULTS

### Clinical and Metabolic Findings

HrC-peptide replacement in diabetic BB/Wor-rat had no effects on body weights, blood glucose levels or daily insulin requirements (Table 1). Plasma hrC-peptide levels were progressively increased in keeping with increasing doses (Table 1). NCV at 2 months of non hrC-peptide replaced diabetic rats was significantly ($p < 0.001$) decreased to 83% of control values. Ten μg of hrC-peptide partially ($p < 0.001$) prevented the NCV deficit, which however still remained significantly ($p < 0.05$) decreased compared to control rats. On the other hand, 100, 500 and 1000 μg of hrC-peptide replacement completely ($p < 0.001$) prevented the nerve conduction defect (Fig 1). Non-hrC-peptide replaced diabetic animals showed a 58% ($p < 0.01$) reduction in $Na^+/K^+$-ATPase activity compared to control rats (Fig 2). Ten and 100 μg hrC-peptide showed no significant prevention of the $Na^+/K^+$-ATPase defect, whereas 500 and 1000 μg completely ($p < 0.05$) prevented the deficit in $Na^+/K^+$-ATPase activity.

### Teased Sural Nerve Fibers

Two months of diabetes revealed structural abnormalities of 20% ($p < 0.001$) of sural nerve myelinated fibers. Ten μg of hrC-peptide had no effect on the frequency of normalcy, whereas 100, 500 and 1000 μg showed significant prevention ($p < 0.01$ for 100 μg; $p < 0.001$ for 500 and 1000 μg) of myelinated fiber pathology, although it was not completely prevented when compared to control rats (Fig 3). The most profound structural change at this stage of type 1 diabetes is the $Na^+/K^+$-ATPase related paranodal swelling

TABLE I Body weights, blood glucose levels, daily insulin requirements and hrC-peptide plasma concentrations at termination of the study (data expressed as mean + SD) in control, sham-operated diabetic and hrC-peptide replaced diabetic BB/Wor-rats

| | Body Weight (g) | Blood Glucose (mmol/l) | Daily Insulin Dose (I.U/day) | HrC-peptide Plasma Concentration ng/ml |
|---|---|---|---|---|
| Control n = 10 | 427 ± 19 | 5.3 ± 0.3 | 0 | 0 |
| Sham-operated n = 7 | 324 ± 21* | 22.5 ± 2.1* | 2.2 ± 0.8* | 0 |
| HrC-peptide 10 μg n = 8 | 325 ± 29*,† | 24.5 ± 3.3*,† | 2.5 ± 0.8*,† | 0 |
| HrC-peptide 100 μg n = 8 | 327 ± 27*,† | 24.3 ± 3.3*,† | 2.9 ± 0.5*,† | 2.8 ± 0.3* |
| HrC-peptide 500 μg n = 8 | 329 ± 19*,† | 23.3 ± 2.8*,† | 2.3 ± 0.8*,† | 9.8 ± 2.5* |
| HrC-peptide 1000 μg n = 8 | 339 ± 23*,† | 23.6 ± 3.4*,† | 2.5 ± 0.7*,† | 31.3 ± 13.9* |

*: $p < 0.001$ vs age-matched non-diabetic control rats; †: No significant difference vs duration-matched sham-operated diabetic rats.

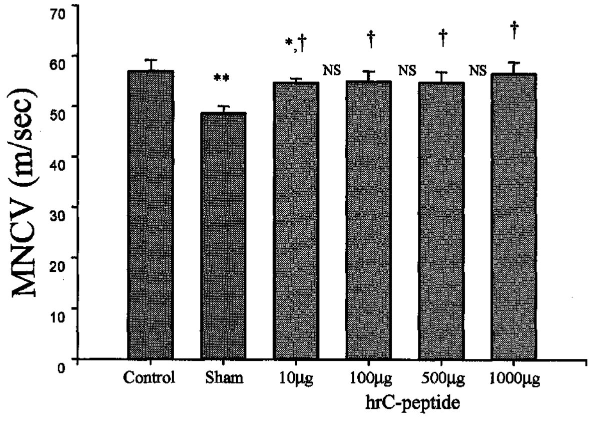

FIGURE 1 Motor nerve conduction velocities (MNCV's) in control, sham-operated diabetic and hrC-peptide replaced BB/Wor - rats at 2 months. *: $p<0.05$, **: $p<0.001$, vs age-matched controls; †: $p<0.001$ vs duration-matched sham-operated BB/W-rats, NS: non-significant.

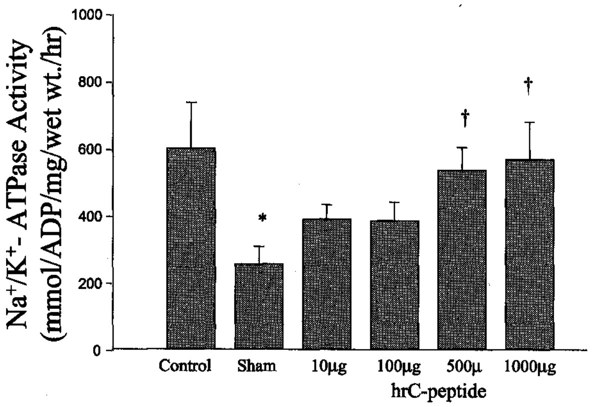

FIGURE 2 $Na^+/K^+$-ATPase activity in sciatic nerves from 2 month control, sham-operated diabetic and hrC-peptide replaced diabetic rats. *: $p<0.01$, vs age-matched controls; †: $p<0.05$ vs duration-matched sham-operated BB/W-rats.

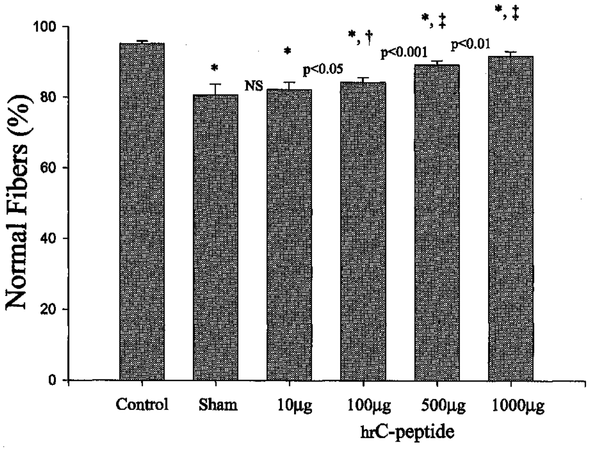

FIGURE 3 Frequencies of structurally normal myelinated fibers in the sural nerve of control, sham-operated diabetic and hrC-peptide replaced diabetic BB/Wor - rats. *: $p<0.001$, vs age-matched control; †: $p<0.01$, vs duration-matched sham-operated BB/W-rats; ‡: $p<0.001$, vs duration-matched sham-operated BB/W-rats, NS: non-significant. The p-values of differences between hrC-peptide replaced groups are indicated.

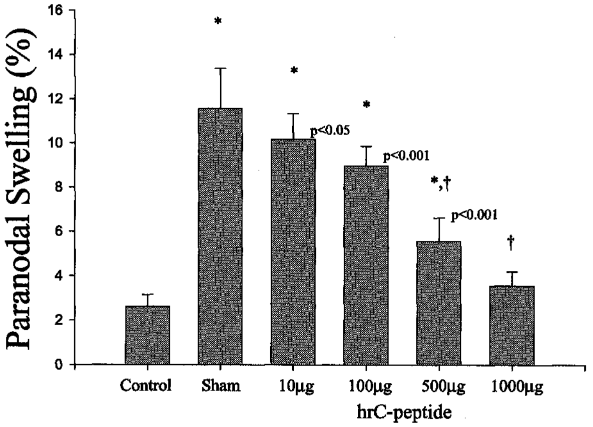

FIGURE 4 Frequencies of the earliest identifiable structural change, paranodal swelling, in control, sham-operated diabetic and hrC-peptide replaced diabetic rats at 2 months. *: $p<0.001$, vs age-matched controls, †: $p<0.001$, vs duration-matched sham-operated BB/W-rats. The p-values of differences between hrC-peptide replaced groups are indicated.

[4,14]. This was increased almost 5-fold ($p<0.001$) in non-hrC-peptide replaceded diabetic rats, but was prevented by 52 and 70% in 500 and 1000 μg hrC-peptide replaced animals respectively (both $p<0.001$; Fig 4), although these values were not normal ($p<0.001$) (Fig 4). Paranodal swelling is followed by paranodal demyelination [4,12], which was exhibited by 1.8% of fibers in non-hrC-peptide replaced diabetic rats (Fig 5). Ten and 100 μg of hrC-peptide had no effect on paranodal demyelination, whereas 500 and 1000 μg fully ($p<0.001$) prevented paranodal demyelination (Fig 5).

Axonal degeneration, comprised of excessive myelin wrinkling, reflecting axonal atrophy, and Wallerian degeneration, was increased 3-fold ($p<0.001$) in non-treated diabetic rats (Fig 6). It was progressively prevented by increasing doses of hrC-peptide replacement and was completely

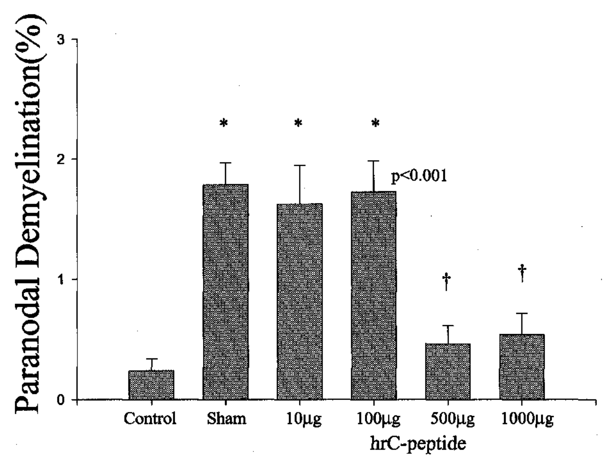

FIGURE 5 Paranodal demyelination in 2 month control, sham-operated diabetic, hrC-peptide replaced diabetic rats. *: $p < 0.001$, vs age-matched controls, †: $p < 0.001$, vs duration-matched sham-operated diabetic BB/W-rats.

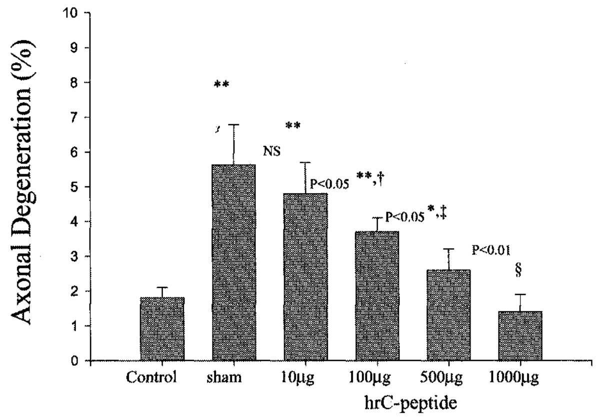

FIGURE 6 Axonal degeneration (sum of frequencies of excessive myelin wrinkling and Wallerian degeneration) in control, sham-operated diabetic and hrC-peptide replaced diabetic BB/Wor – rats. *: $p < 0.01$, **: $p < 0.001$, vs age-matched controls, †: $p < 0.05$, ‡: $p < 0.01$, §: $p < 0.001$, vs duration-matched sham-operated diabetic BB/W-rats, NS: non-significant. The p-values of differences between hrC-peptide replaced groups are indicated.

($p < 0.001$) prevented by 1000 μg of hrC-peptide (Fig 6).

Increasing doses of hrC-peptide promoted nerve fiber regeneration in the sural nerve, the frequencies of which were significantly ($p < 0.001$) increased in 500 and 1000 μg replaced animals (Fig 7).

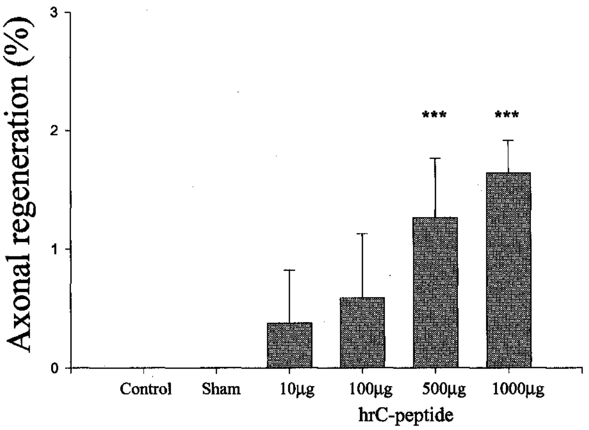

FIGURE 7 Frequencies of regenerated myelinated fibers in control, sham-operated diabetic and hrC-peptide replaced diabetic rats at 2 months. ***: $p < 0.001$, vs age-matched controls and sham-operated diabetic BB/W-rats.

### DISCUSSION

The present data demonstrate a dose related prevention of functional, metabolic and structural abnormalities of early DPN in type 1 diabetic BB/Wor-rats by hrC-peptide. These findings are similar to those previously reported following replacement with type II rat C-peptide [4]. Similar to the findings in this study, parameters such as normalcy and paranodal swelling were not fully prevented in hyperglycemic rats even with the highest replacement dose of hrC-peptide in the present study. We have previously suggested, by comparing the DPN in the type 1 C-peptide deficient diabetic BB/Wor-rat with that in the normal C-peptidemic type 2 BB/Z-rat [12], that DPN in type 1 rats is caused by a hyperglycemic component and a C-peptide responsive component. Since activation of the polyol pathway is also associated with these early structural changes [15] and is not corrected by C-peptide replacement [4], it is possible that the residual structural abnormalities in the present study reflect polyol-pathway induced abnormalities similar to those seen in the milder DPN of type 2 BB/Z-rats [12].

The prevention of the $Na^+/K^+$-ATPase defect in diabetic peripheral nerve is in keeping with our previous data [4] and those of others [1,9], and probably accounts for the prevention of the early metabolic NCV defect, which is closely associated with decreased $Na^+/K^+$-ATPase activity [16]. Nodal changes such as axo-glial dysjunction, paranodal demyelination and intercalated internodes are characteristic of type 1 DPN in human and animal models but do not occur in type 2 DPN [12,17,18]. The complete prevention of paranodal demyelination by 500 and 1000 μg hrC-peptide is consistent with previous findings, showing complete prevention of the preceding axo-glial dysjunction and paranodal demyelination in the type 1 rat model [4]. Sugimoto et al [19] have demonstrated that the insulin receptor co-localizes with paranodal axo-glial junctions in peripheral nerve. We [19] and others [20] have suggested that insulin may have a regulatory effect on nodal tight junction integrity. This is consistent with C-peptide's insulinomimetic and preventive effects on disruption of paranodal axo-glial junctions and paranodal demyelination. The dose related increase in nerve fiber regeneration is in agreement with our previous study [4] and is possibly related to C-peptide's corrective effect on IGF-1 expression in peripheral nerve [21]. Although not examined in the present study, C-peptide has an ameliorating effect on endothelial NO [22]. Such a beneficial effect on NO and microcirculation is likely to improve endoneurial blood flow, hence contributing to the prevention of DPN.

The amino-acid sequence of C-peptide shows considerable species variations [8]. It has been suggested that the conserved midportion mediates its biological effect [1]. However, this has not been confirmed by others [8,11], who instead have shown that the C-terminal pentapeptide is responsible for C-peptide activity. The active C-terminal pentapeptide differs by three amino-acids between rat and man. In the present study approximately $10^4$ higher concentrations of hrC-peptide was necessary to achieve the same effects as those reported with rat II C-peptide (75 nmol/kg body weight/day) [4], which may be due to the only partially conserved regions of the C-terminal which possesses the receptor/ligand type interaction [8].

In summary the present study has shown dose-dependent effects of hrC-peptide on early DPN in the type 1 diabetic BB/Wor-rat, demonstrating its preventative effect and cross-species activity which is likely mediated by the partially conserved sequence of the biologically active C-terminal.

*Acknowledgement*

This study was supported by a grant from Schwarz Pharma AG, Monheim, Germany.

### *References*

[1] Ido Y, Vindigni A, Chang K, Stramin L, Chance R, Heath WF, DiMarchi RD, DiCera E, and Williamson JR (1997). Prevention of vascular and neural dysfunction in diabetic rats by C-peptide. *Science* 777:563-566.

[2] Johansson B-L, Borg K, Fernquist-Forbes E, Odergren T, Remahl S, Wahren J (1996). C-peptide improves autonomic nerve function in patients with type 1 diabetes. *Diabetologia* 39:687-695.

[3] Johansson B-L, Borg K, Fernquist-Forbes E, Kernell A, Odergren T, Wahren J (2000). Beneficial effects of C-peptide on incipient nephropathy and neuropathy in patients with type 1 diabetes–a three month study. *Diabetic Medicine* 17:181-189.

[4] Sima AAF, Zhang W, Sugimoto K, Henry D, Li Z-G, Wahren J, Grunberger G (2001): C-peptide prevents and improves chronic type 1 diabetic polyneuropathy in the BB/Wor-rat. Diabetologia 44:889-897.

[5] Li Z-G, Qiang X, Sima AAF, Grunberger G (2001). C-peptide attenuates protein tyrosine phosphatase activity and enhances glycogen synthesis in L6 myoblasts. Biochem Biophys Res Com 280:615-619.

[6] Grunberger G, Qiang X, Li Z, Mathews ST, Sbrissa D, Shisheva A, Sima AAF (2001): Molecular basis for the insulinomimetic effects of C-peptide. Diabetologia (in press).

[7] Jensen ME, Messina EJ (1999): C-peptide induces a concentration-dependent dilatation of skeletal muscle arterioles only in the presence of insulin. Am. J. Physiol. 276;H1223-H1228

[8] Rigler R, Pramanik A, Jonasson P, Kratz G, Jansson OT, Nygren P-Å, Ståhl S, Ekberg K, Johansson B-L, Uhlén S, Uhlén M, Jörnvall H, and Wahren J· (1999). Specific binding of proinsulin C-peptide to human cell membranes. *Proc. Natl. Acad. Sci.* 96:13318-13323.

[9] Ohtomo Y, Aperia A, Sahlgren B, Johansson B-L, Wahren J (1996). C-peptide stimulates rat renal tubular $Na^+$, $K^+$ ATPase activity in synergism with neuropeptide Y. *Diabetologia* 39:199-205.

[10] Forst J, Kunt T, Pohlmann T, Goitom K, Engelbach M, Beyer J, Pfützner A (1998): Biological activity of C-peptide on the skin microcirculation in patients with insulin-dependent diabetes mellitus. J Clin Invest. 101: 2036-2041.

[11] Wahren J, Ekberg K, Johansson J, Henriksson M, Pramanik A, Johansson B-L, Rigler R, Jörnvall H

(2000). Role of C-peptide in human physiology. *Am. J. Physiol.* 278:759-768.

[12] Sima AAF, Zhang W, Xu G, Sugimoto K, Guberski D, Yorek MA (2000). A comparison of diabetic polyneuropathy in type II diabetic BBZDR/Wor-rats and type I diabetic BB/Wor-rats. *Diabetologia* 43:786-793.

[13] Yorek MA, Wiese TJ, Davidson EP, Dunlap JA, Stefani MR, Conner CE, Lattimer SA, Kamijo M, Greene DA, Sima AAF (1993): Reduced motor nerve conduction velocity and Na+-K+-ATPase activity in rats maintained on L-fucose diet. Reversal by myo-inositol supplementation. Diabetes 42:1401-1406.

[14] Sima AAF, Brismar T (1985). Reversible diabetic nerve dysfunction. Structural correlates to electrophysiological abnormalities. *Ann. Neurol.* 18:21-29.

[15] Sima AAF, Prashar A, Zhang W-X, Chakrabarti S, Greene DA (1990). Preventive effect of long-term aldose reductase inhibition (Ponalrestat) on nerve conduction and sural nerve structure in the spontaneously diabetic BB-rat. *J. Clin. Invest.* 85:1410-1420.

[16] Sima AAF, Sugimoto K (1999). Experimental diabetic neuropathy: an update. *Diabetologia* 42:773-788.

[17] Sima AAF, Nathaniel V, Bril V, McEwen TAJ, Greene DA (1988). Histopathological heterogeneity of neuropathy in insulin-dependent and non-insulin-dependent diabetes, and demonstration of axo-glial dysjunction in human diabetic neuropathy. *J. Clin. Invest.* 81:349-364.

[18] Sima AAF, Lattimer SA, Yagihashi S, Greene DA (1986). "Axo-glial dysjunction": A novel structural lesion that accounts for poorly reversible slowing of nerve conduction in the spontaneously diabetic BB-rat. *J. Clin. Invest.* 77:474-484.

[19] Sugimoto K, Murakawa Y, Zhang W, Xu G, Sima AAF (2000): Insulin receptor in rat peripheral nerve: its localization and alternatively spliced isoforms. Diabetes Metab Res Rev. 16:354-363.

[20] Freychet P (2000): Insulin receptor and insulin actions in the nervous system. Diabetes Metab Res Rev. 16: 390-392.

[21] Sima AAF, Zhang W, Xu G, Wahren J, Sugimoto K (1999): Long term effect of C-peptide treatment on type 1 diabetic neuropathy. Diabetes 48. Supple 1, A152 (abstract).

[22] Forst T, De la Tour DD, Kunt T, Pfützner A, Goitom K, Pohlmann T, Schneider S, Johansson BL, Wahren J, Lobig M, Engelbach M, Beyer J, Vague P (2000): Effects of proinsulin C-peptide on nitric oxide, microvascular blood flow and erythrocyte $Na^+$, $K^+$-ATPase activity in diabetes mellitus type 1. Clin Sci. 98:283-290.

